# Snord 3A: A Molecular Marker and Modulator of Prion Disease Progression

**DOI:** 10.1371/journal.pone.0054433

**Published:** 2013-01-21

**Authors:** Eran Cohen, Dana Avrahami, Kati Frid, Tamar Canello, Ephrat Levy Lahad, Sharon Zeligson, Shira Perlberg, Joab Chapman, Oren S. Cohen, Esther Kahana, Iris Lavon, Ruth Gabizon

**Affiliations:** 1 Department of Neurology, Hadassah Medical Center, The Agnes Ginges Center of Human Neurogenetics, Jerusalem, Israel; 2 Medical Genetics Institute, Shaare Zedek Medical Center, Jerusalem, Israel; 3 Hebrew University-Hadassah Medical School, Jerusalem, Israel; 4 Department of Neurology, Sheba Medical Center, Sackler Faculty, Tel Aviv University, Israel; 5 Department of Neurology, Barzilai Medical Center, Ashkelon, Israel; Pasteur Institute of Lille, France

## Abstract

Since preventive treatments for prion disease require early identification of subjects at risk, we searched for surrogate peripheral markers characterizing the asymptomatic phases of such conditions. To this effect, we subjected blood mRNA from E200K PrP CJD patients and corresponding family members to global arrays and found that the expression of Snord3A, a non-coding RNA transcript, was elevated several times in CJD patients as compared to controls, while asymptomatic carriers presented intermediate Snord3A levels. In the brains of TgMHu2ME199K mice, a mouse model mimicking for E200K CJD, Snord 3A levels were elevated in an age and disease severity dependent manner, as was the case for brains of these mice in which disease was exacerbated by copper administration. Snord3A expression was also elevated in scrapie infected mice, but not in PrP^0/0^ mice, indicating that while the expression levels of this transcript may reflect diverse prion etiologies, they are not related to the loss of PrP^C^’s function. Elevation of Snord3A was consistent with the activation of ATF6, representing one of the arms of the unfolded protein response system. Indeed, SnoRNAs were associated with reduced resistance to oxidative stress, and with ER stress in general, factors playing a significant role in this and other neurodegenerative conditions. We hypothesize that in addition to its function as a disease marker, Snord3A may play an important role in the mechanism of prion disease manifestation and progression.

## Introduction

Prion diseases, characterized by the accumulation of the oxidized and misfolded PrP^Sc^, are late onset fatal neurodegenerative conditions that can present as sporadic, transmissible, and inherited etiologies [1]. Indeed, the notion that protein misfolding is associated with brain degeneration was first shown for PrP^Sc^ in prion diseases [2], but since then a “prion like” paradigm has been also described for conditions such as Alzheimer’s and Parkinson [3], and their associated proteins, Tau, α-synuclein and Aβ [4], [5]. It is well recognized today that in all these conditions progressive irreversible brain damage is established long before disease signs becomes apparent [6], implying that effective intervention should be of preventive nature, following the identification of subjects prone to contract neurodegenerative conditions. This prophylactic strategy depends on the possibility to identify subjects at risk by peripheral testing while still at the asymptomatic stage. Indeed, PrP^Sc^, the ultimate marker of prions, can be detected in accessible peripheral tissues such as blood after sophisticated amplification procedures [7], but these methods are non-quantitative enough to be applied on asymptomatic human subjects. Testing levels of disease markers in accessible tissues is imperative, since such examination will need to be repeated periodically through the life span of the individual as well as during preventive treatments, once these are developed.

As opposed to sporadic and transmissible prion diseases, individuals at risk to develop genetic prion disease, such as Creutzfeldt-Jacob disease (gCJD), are easily recognized since both patients and asymptomatic family members carry dominant pathogenic mutations in the PrP gene [8]–[9]. Asymptomatic carriers, which will most probably develop the disease at individual time points in their future, may well express disease markers in an age and/or disease progression dependent manner. Once identified for the genetic disease, these markers may also be evaluated in individuals incubating other forms of prion disease, such as those who were exposed to BSE infected meat, or to contaminated blood [10].

The largest focus of gCJD was identified among Libyan Jews carrying a mutation at PrP codon 200 (substitution of lysine for glutamate, also denominated E200K CJD) [11], [12], [13]. This same mutation was described in other communities around the world [14] and constitutes the most prevalent PrP mutation. In addition, E200K CJD is the familial prion disease most similar in its clinical presentation to sporadic CJD [15], [16], suggesting features described for this disease form may also relate to other CJD patients and at risk subjects.

In the search of new prion disease markers, we subjected blood mRNA form E200K patients, carriers and non-carriers family controls to Global expression studies, a methodology used for the search of markers and the investigation of disease mechanism in many diseases [17], [18]. Subsequently, we validated the results for candidate genes in additional humans samples by Real-Time PCR and then tested the levels of such transcripts in the brains of TgMHu2ME199K mice [19] of different ages, to establish whether the expression of the candidate transcripts represents age dependent disease progression. TgMHu2ME199K mice constitute a model for E200KCJD patients, since they present an age dependent fatal progressive disease, characteristic PrP neuropathology and disease transmission to wt animals [19]. Using this strategy, we identified Snord3A, a non-coding RNA transcript [20], as a marker for prion disease progression. Snord 3A expression was elevated in the blood of CJD patients as compared to non-carrier controls, while healthy carriers presented intermediate expression levels. Consistent with the human studies, Snord3A levels in the brains of TgMHu2ME199K mice were strongly elevated in an age and disease dependent fashion. Snord3A levels were also elevated in scrapie infected mice, indicating this transcript is not only associated with genetic prion disease but also relates to other etiological prion disease presentations in which misfolded protease resistant PrP is accumulated. Indeed, we show here that the elevation of Snord3A expression was consistent with the activation of at least one arm of the unfolded protein response (UPR) system [21].

## Results

### Identification of E200K CJD Disease Specific Markers

**Figure 1:**
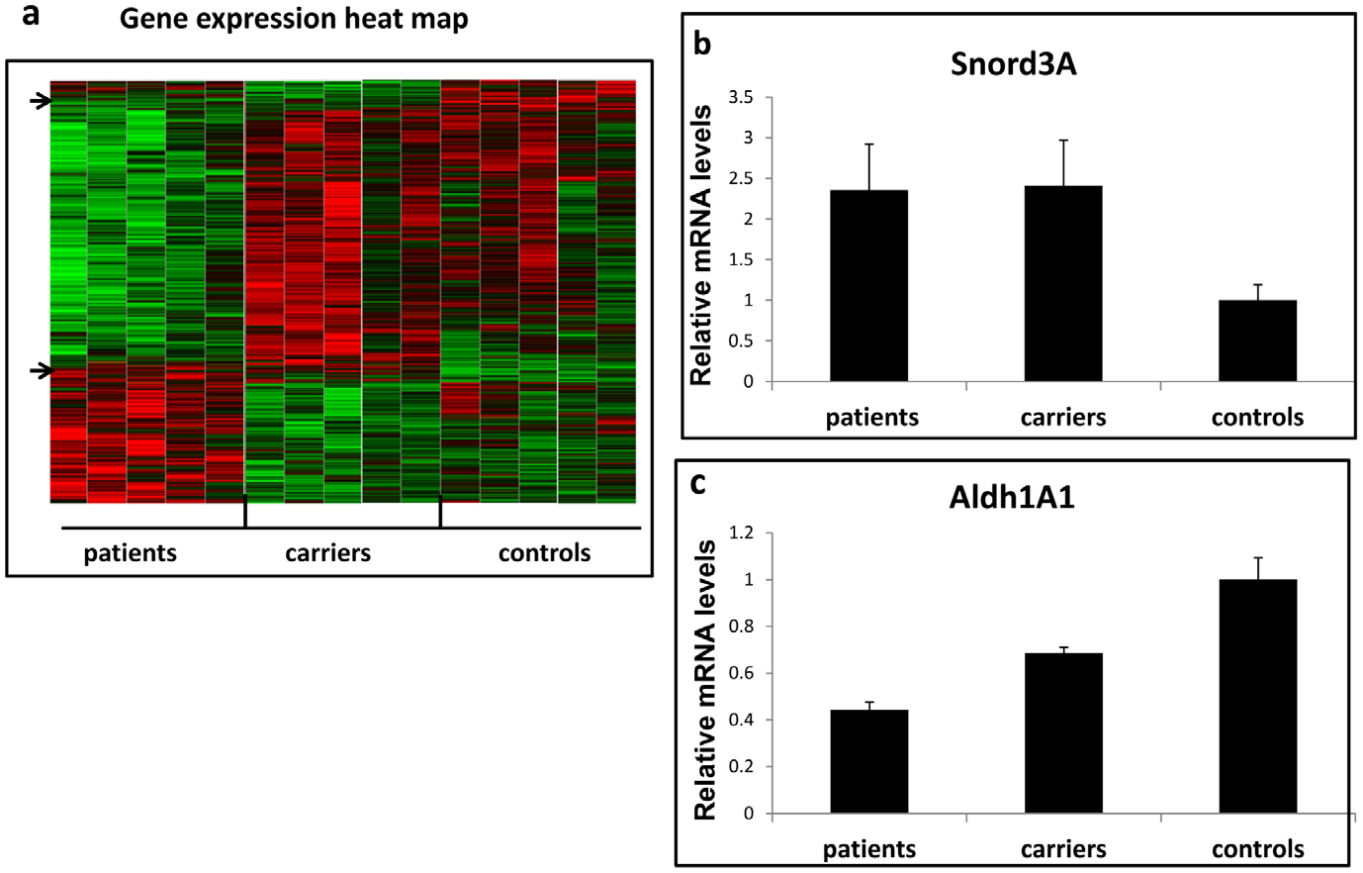
Identification of Snord3A and Aldh1A1 as disease specific markers. a: Gene expression heat map representing the levels of transcripts expressed in blood from E200KCJD patients, carriers and non-carriers control. b: Snord3A expression level in microarrays of patients and healthy mutation carriers as compared to non-carrier controls. C: Aldh1A1 expression level in patients and healthy mutation carriers as compared to non-carrier controls (P value <0.005).

Global gene expression [22] was assayed (using Affymetrix Hu Gene 1.0 ST arrays, see Materials and Methods) in (total) RNA extracted from white blood cells of E200K CJD patients, healthy carriers and related non-carrier controls (5 in each group). The gene expression analysis revealed 427 genes that were differentially expressed (/p value≤0.05)) in E200KCJD patients and carriers compared to non-carrier controls. Figure 1a presents a gene expression heat map, demonstrating significant differences in the gene expression patterns of sick and non-sick individuals, and yet a subtle but visible difference between the patterns of controls and the mutant subjects (arrows). In E200K CJD patients, small nucleolar RNA3A (Snord3A), was the most up-regulated gene: elevated by 3 fold in patients and E200K carriers as compared to related non-carrier controls (P = 0.004) (Fig. 1b). Aldehyde dehydrogenase 1 (ALdh1A1) was the most down-regulated gene with a 34% decrease in expression in E200K carriers (P = 0.0048), and an even greater reduction (59%) in CJD patients, compared to related non-carrier controls (P<0.0001) Fig. 1c). A list of the 10 most differentially expressed transcripts between patients and controls is shown in Table 1.

**Table 1:** Differential gene expression profile of E200KCJD patients and carriers compared with non carriers control.

P value	Fold-change	Gene	Gene symbol
0.004051	3.04209	Small nucleolar RNA, C/D box 3A	SNORD3A
0.009815	1.94306	Tubulin, beta 2A class IIa	TUBB2A
0.000109	−1.9012	Aldehyde dehydrogenase 1 family, member A1	ALDH1A1
0.00634	1.74713	Histone cluster 1, H4d	HIST1H4D
0.000718	−1.70155	Male-specific lethal 3 homolog (Drosophila) pseudogene 1	MSL3P1
0.047058	−1.67503	Killer cell lectin-like receptor subfamily C, member 2	KLRC2
0.009088	1.63774	Keratin 73	KRT73
0.020534	1.62706	Small nucleolar RNA, H/ACA box 16B	SNORA16B
0.027231	−1.61474	Chromosome 7 open reading frame 58	C7orf58
0.045771	−1.546	Steroid 5 alpha-reductase 3	SRD5A3

**Figure 2:**
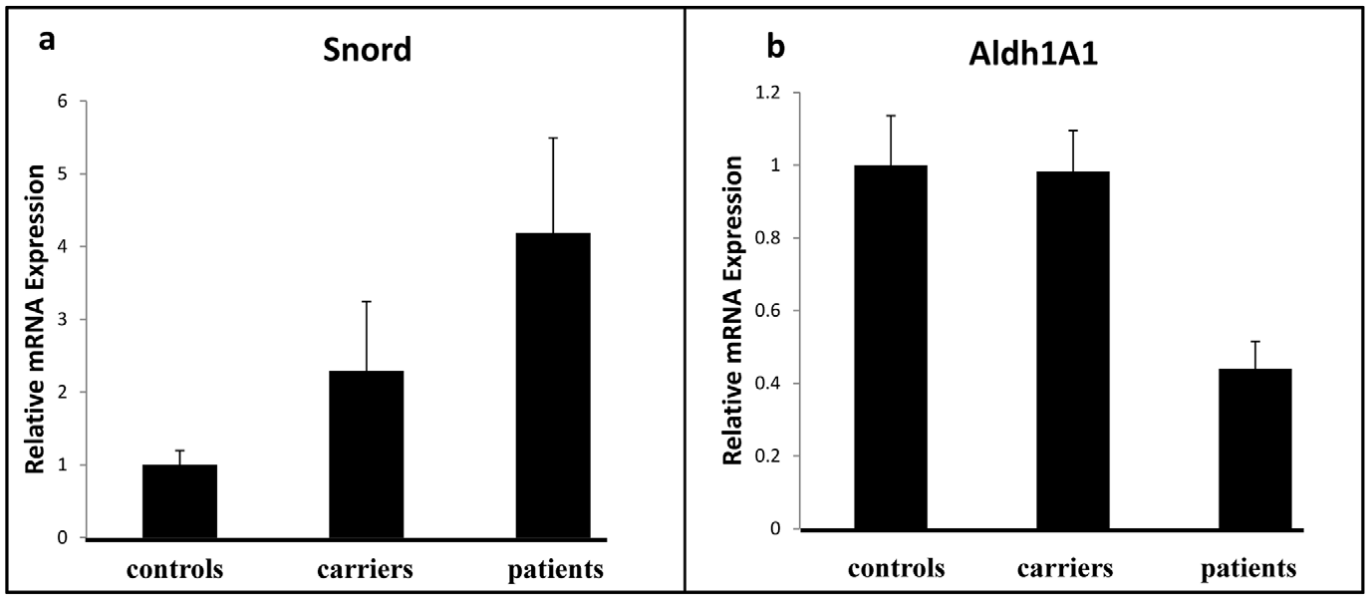
Validation of microarray markers by Quantitive Real Time PCR. Total RNA was extracted from E200KCJD patients, carriers and non-carrier controls were reverse transcribed into cDNA and amplified for Snord3A and control genes as described in the Materials and Methods section. Relative expression levels were normalized in reference to HPRT. a: Snord3A expression levels. b: Aldh1A1 expression levels (P value = 0.005 ).

To validate the microarray results, expression levels of Snord 3A (the most elevated transcript) and ALdh1A1 (the most reduced) were tested in patients, healthy carriers and related non-carrier controls using real-time PCR. Snord 3A is a non-coding RNA transcript from the box C/D SnoRNA family [23]. As for today, several functions have been tentatively attributed to SnoRNA transcripts [23], [24], in particular as regulators of stress response [25]. As for ALDH1, its function is well established as the enzyme responsible of degrading 4-hydroxynonenal (HNE), the most toxic product of lipid peroxidation [26]. Reduced expression and activity of ALDH1 may be consistent with diminished protection against toxic lipid degradation [27]. Results from these studies (Fig. 2a) were mostly consistent with the microarray results and show that while Snord3A was significantly elevated in all patients tested as compared to non-carrier controls, healthy mutation carriers presented in this case average intermediate levels between controls and patients. As for the ALdh1 levels (most down-regulated gene), while a significant difference in expression levels was observed between patients and controls, in this case the levels in carriers were more similar to those of controls (Fig. 2b). These results suggest that E200K CJD patients may suffer from disruptions in pathways protecting against oxidative stress [28], [29], [30].

### Validation of Human Results in the TgMHu2ME199K Mouse Model

**Figure 3:**
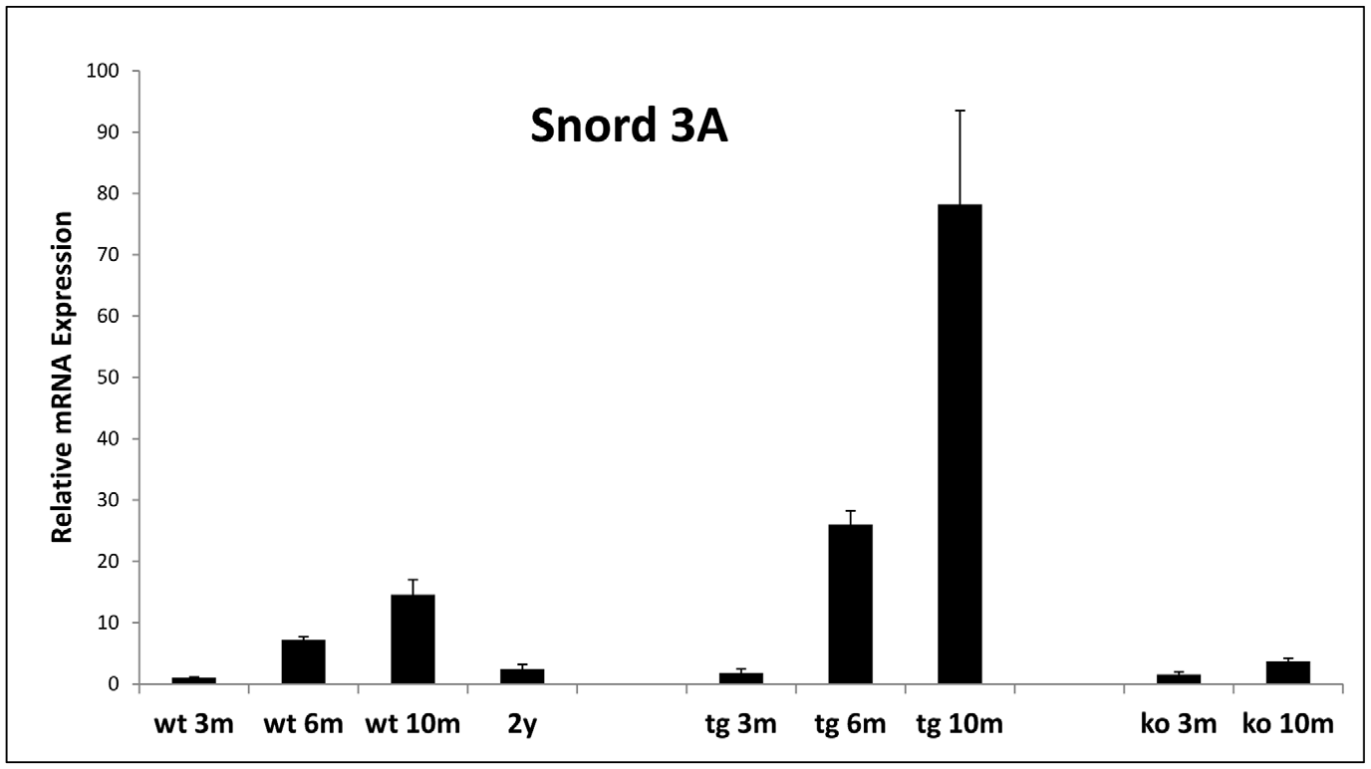
Elevation of Snord3aA expression in Tg MHu2M E199K mice brains is disease dependent. Total RNA extracted from brains of TgMHu2ME199K (tg), PrP ^0/0^ (ko) and wt mice, od designated ages were reverse transcribed into cDNA and amplified by Real Time PCR as described in Materials and methods. Each group comprised 4–7 mice. Gene expression changes are depicted in relevance to those measured at 3 months old wt mice. Relative expression levels were normalized in reference to UBC. (** P value <0.001).

While it may take years of repeated testing in aging human carriers to establish whether Snord 3A or ALdh1 expression levels represent markers of advancing prion disease in humans, and to study more about the mechanistic correlation of these markers to prion disease progression in the brain, we tested their expression in the brains of TgMHu2ME199K mice [19]. These transgenic mice express a chimeric mouse/human (TgMHu2M) E199KPrP on a null PrP background, and were shown by us to contract and subsequently succumb to prion disease in an age dependent manner [19]. In addition, TgMHu2ME199K mice accumulate disease related PrP, as shown both by PrP pathology and immunoblotting, and their brains also transmit disease to wt mice. Brain RNAs from wt, PrP^0/0^ and TgMHu2ME199K mice obtained at preclinical (3 months) and at additional time points were tested by Real time PCR for the expression levels of ALDH1 and Snord3A (Figure 3). The expression of ALDH1 in the mice brains was mostly uninformative (not shown) in all cases, however this was not the case for Snord 3A, the ncRNA transcript. While in all young mice (3 month old) Snord3A levels were similar, it was only in the TgMHu2ME199K mice that Snord3A levels were elevated in a significant age dependent manner. Indeed elevation of Snord3A levels in these mice was consistent with the age and disease dependent accumulation of PK resistant PrP, barely detected at 3 months old mice and significantly increasing thereafter [19]. The fact that Snord3A expression did not increase in the PrP ablated mice, further indicates that this transcript may in some way signal for the levels of the misfolded protein accumulation, as opposed to reduced presence of the normal PrP. In addition, the mice results are consistent with the notion that Snord3A expression levels in human blood may serve as a marker for disease progression.

### Snord Expression is Elevated in TgMHu2ME199K Mouse Embryonic Fibroblasts

**Figure 4:**
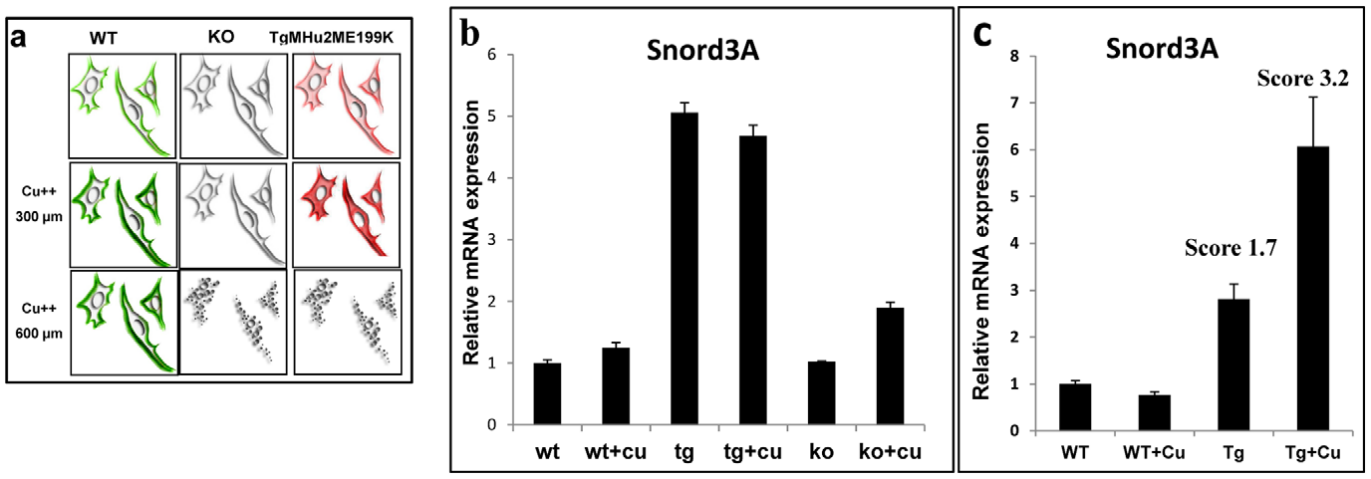
Elevation of Snord3A expression in Tg MHu2M E199K MEFs and in copper treated mice. Total RNA from brains of TgMHu2ME199K and wild-type mice as described, as well as from designated MEFs were amplified for Snord3A by Real time PCR. Relative RNA expression levels were normalized in reference to UBC and *β-actin* (Respectively). a: Scheme of accumulation of PrP and MEF survival during copper treatment (as described in [32]). b: Snord3A levels in designated MEFs (wt: cells from wt mice; tg: cells from TgMHu2ME199K mice; KO: cells from PrP^0/0^ mice. c: Snord3A levels in TgMHu2ME199K and wild-type mice brain after 75 days of copper treatment versus age-matched control (** P value <0.001).

Next, we tested the levels of Snord3A in mouse embryonic fibroblasts **(**MEFs), generated from TgMHu2ME199K, wt and PrP^0/0^ 14 days mouse embryos. These cells are considered of primary nature, however they preserve the ability to divide for several generations [31]. We have recently shown that TgMHu2ME199K MEFs accumulate PrP both on the membrane and intracellulary, as opposed to wt cells which only present PrP on the outer membrane [32]. The cartoon in Figure 4a describes the properties of these cells vis a vis PrP expression and copper toxicity. Copper induced the overexpression of PrP in both wt and TgMHu2ME199K MEFs, however wt MEFs were resistant to copper toxicity, as opposed to mutant and PrP ablated cells. In this work, we tested the expression of Snord3A by Real Time PCR in wt, PrP^0/0^ and TgMHu2ME199K MEFs following their culturing in the presence and absence of 300 µm copper, a non-toxic copper concentration for all cells. Figure 4b shows that Snord3A expression levels were elevated only in the TgMHu2ME199K cells and that no further elevation was observed when these cells were cultured in the presence of 300 µm copper, regardless of the overexpression of PrP, outside and inside the cells. These results suggest that the elevation in Snord3A levels in the TgMHu2ME199K cells may mark the intrinsic intracellular accumulation of mutant PrP. Further elevation of Snord3A expression may requires an additional pathological event. Such event, however, cannot be related to the loss of PrP^C^, since both PrP ablated cells and mice didn’t not show an increase in Snord3A levels.

### Elevated Levels of Snord3A in MHu2ME199K Tg Mice and Cells Following Addition of Copper

As opposed to MEFs in which both TgMHu2ME199K and PrP^0/0^ cells were extremely sensitive to copper toxicity, copper administration significantly accelerated disease progression only in the TgMHu2ME199K [32]. To further investigate whether Snord3A expression represents prion disease progression as opposed to general stress, we tested the levels of Snord3A in RNA samples from brains of wt and TgMHu2ME199K mice following administration of copper for 75 days, starting at 3 months of age [32]. Figure 4c shows that concomitantly with the copper induced disease acceleration (disease score [19] elevated from 1.7 in untreated to 3.2 in copper treated mice), the levels of Snord3A in the TgMHu2ME199K mice were elevated about two fold as compared to the untreated mice. No effect on Snord3A levels was observed in the healthy treated wt mice, as well as on the slightly affected PrP^0/0^ mice (not shown). These results further indicate that the levels of Snord expression represent the progression and severity of prion disease manifestation.

### Snord3A Expression in Brains of Scrapie Infected Mice

**Figure 5:**
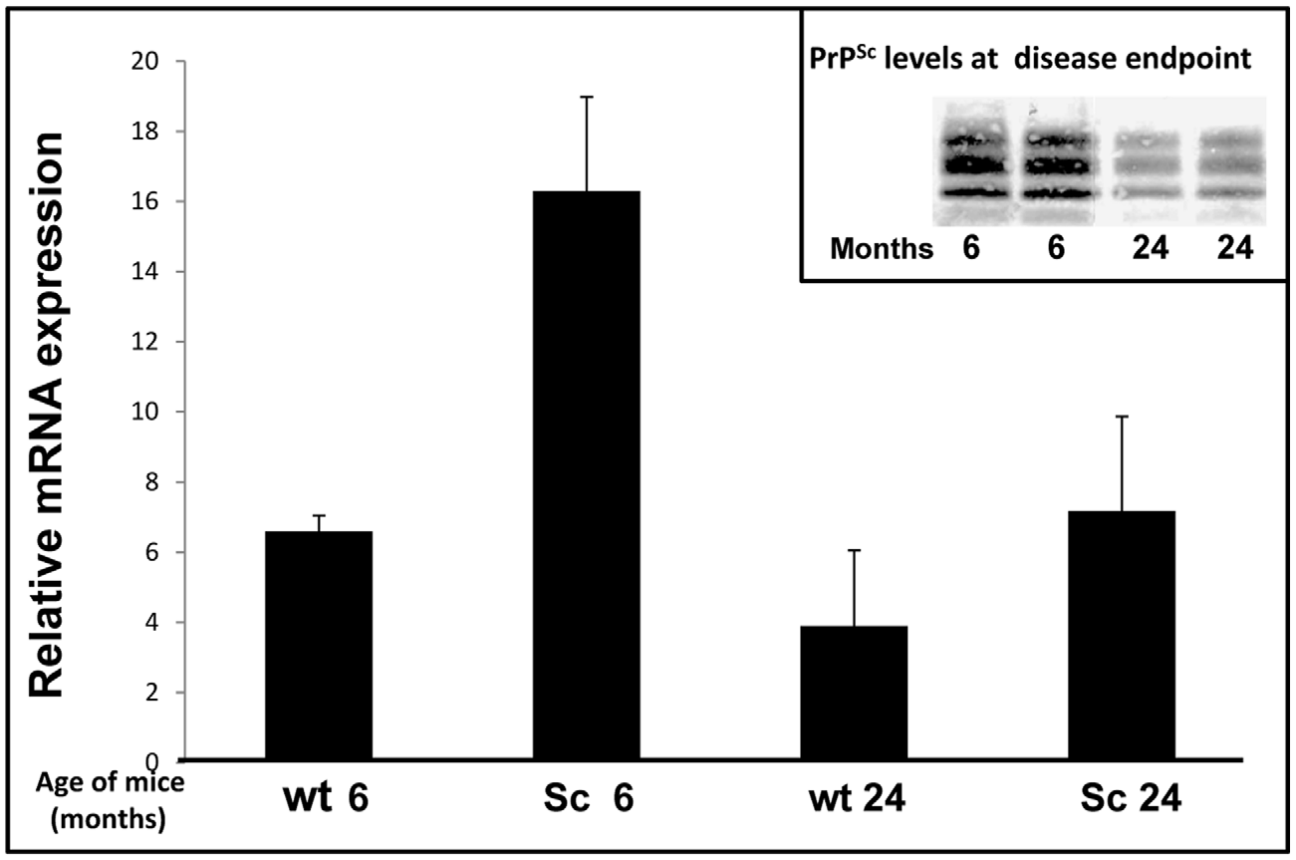
Expression of Snord3A in scrapie-infected brain’s. Main picture: Total RNA from brains of scrapie infected mice (6 and 24 months old mice, 4 in each group) and age matched wt controls were amplified for Snord3A by real time PCR as described. Relative RNA expression levels were normalized in reference to UBC. Relative Snord3A expression level in scrapie infected mice is indicated by fold change versus age-matched controls. Insert: Immunoblotting of PrP^Sc^ in brains of infected mice (2 young, two old). (** P value <0.001).

To test whether expression of Snord3A constitutes a marker of prion disease etiologies others then genetic, we tested the levels of Snord 3A expression in mice infected with the RML scrapie strain at different ages, as compared to age matched uninfected mice. Indeed, we have shown previously that older mice present a different response to prion infection as compared to young mice, as reflected by longer incubation time, milder neuropathological features, and markedly reduced accumulation of PrP^Sc^ [33]. Also the up regulation of inflammatory and stress-response genes upon prion infection [34], [35] was greatly reduced when tested in the mice that were infected at old age [33]. Figure 5 shows a similar response for Snord3A expression levels when compared in brains of sick mice infected either at young or old age. While Snord3A expression was elevated in both scrapie infected groups, the degree of elevation was more pronounced for mice infected at young age (1 month) and succumbing to disease 5 months later, as compared to mice infected at old age (16 months) and succumbing to disease when 2 years old. The insert in the Figure represents the levels of PrP^Sc^ accumulation in the young and older mice, showing a significant excess of the prion protein for the younger mice. Based on all these results, we hypothesize that Snord3A expression levels may reflect an array of prion markers, ranging from levels of PrP^Sc^ accumulation, as well as increased expression of stress response markers, again more elevated at young age [33]. Most important, our results indicate that Snord3A expression constitutes a marker not only of genetic prion disease but also for transmissible prions, indicating a common mechanism of action for both conditions.

### Unfolded Protein Response in TgMHu2ME199K Brains

**Figure 6:**
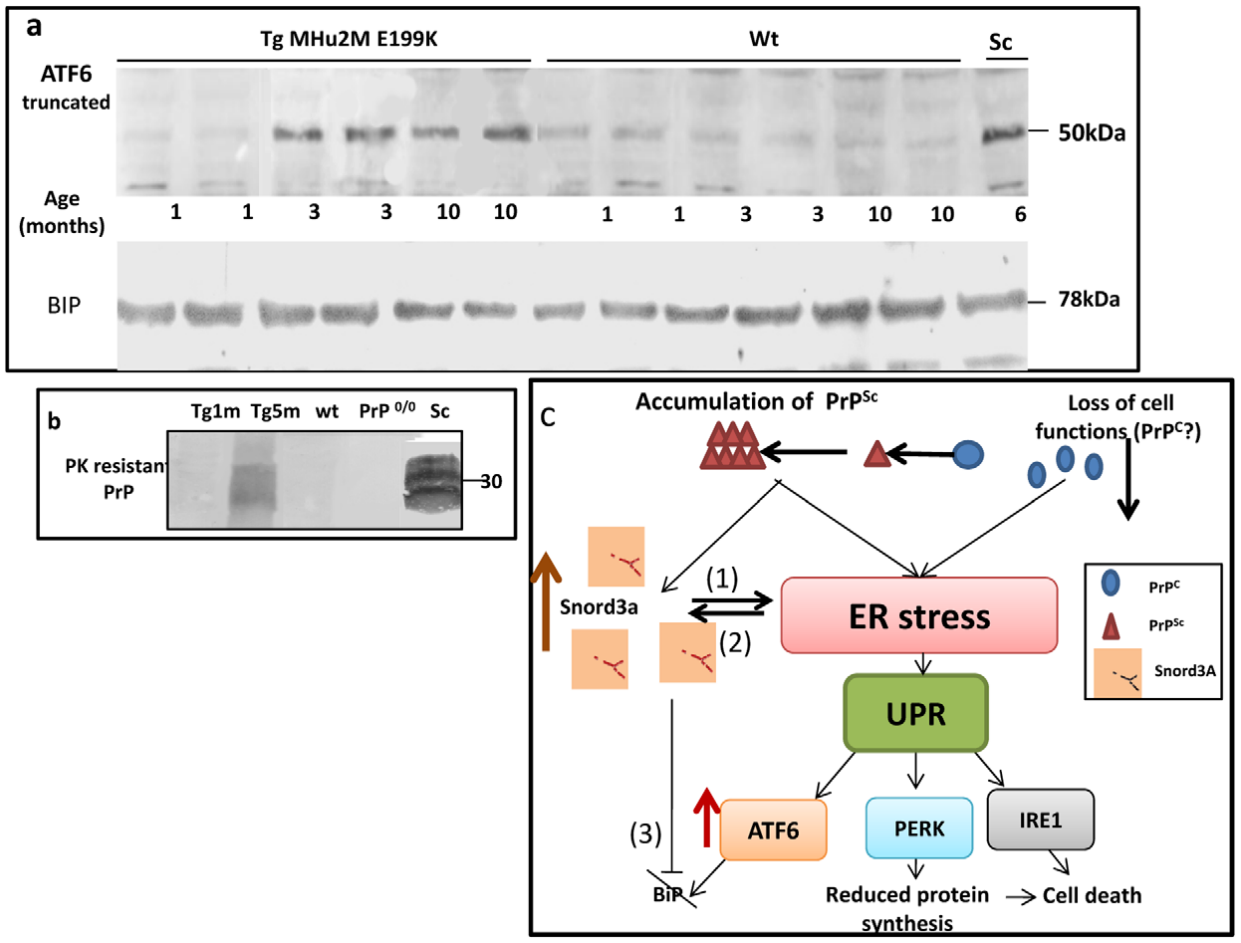
ATF6 is activated in genetic and transmissible prion disease. Brain homogenates from 1, 3, and 10 months old wild-type and TgMHu2ME199K mice and 6 months old RML-infected mice were extracted with sarkosyl and immunoblotted for ATF6 and BiP (a), and PK resistant PrP (b) with antibody as described in Materials and methods. c: Scheme of the UPR system with possible roles for Snord3A.

SnoRNAs transcripts were shown to participate in protein complex formation [23] and also to play a role in oxidative, metabolic or ER stress situations [25], [36]. Indeed, cellular stress may disrupt the functioning of the endoplasmic reticulum (ER), a critical organelle for protein quality control, leading to accumulation of misfolded proteins and induction of the unfolded protein response (UPR) [21], a homeostatic signaling network that orchestrates the recovery of ER function when possible and induces apoptosis when such recovery fails. ER response pathways such as the UPR are believed to play an important role in neurodegenerative conditions in general [37]. In prion diseases, the role of the UPR system is still unclear. While it was recently shown that accumulation of PrP^Sc^ in disease causes persistent translational repression of global protein synthesis by eIF2α-P [38], representing the PERK arm of the UPR, other investigators could not find differences in the expression of BIP (immunoglobulin heavy chain-binding protein, also known as GRP78) [21], an important chaperone resulting from UPR activation [39](see scheme in Figure 6c). We now tested the levels of activated ATF6 (activating transcription factor), a major UPR protein responsible for the up regulation of chaperones such as BIP [40] in the brains of TgMHu2ME199K, scrapie infected and wt mice. Our results show that in the older (sick) TgMHu2ME199K mice brains as well as in scrapie infected brains, truncated ATF6 (representing activated ATF6) was significantly accumulated (Fig. 6a), concomitantly with the accumulation of PK resistant PrP (Fig. 6b). However, and in accordance with previous results [39], the activation of ATF6 in these prion models didn’t result in more production of chaperons such as BIP (Fig. 6a), suggesting the correcting arm of this UPR pathway is initially activated but then blocked. Whether elevated Snord3A expression relates to this pathway by triggering the UPR response or by blocking the subsequent chaperon activation remains to be elucidated.

## Discussion

We have shown by microarray studies of blood samples from E200K CJD patients and controls that Snord3A may serve as a marker of this genetic prion disease. Next, the levels of this transcript were measured in brains of TgMHu2ME199K mice, a line modeling for E200K CJD, and results of these experiments show that Snord3A may serve not only as an indicator of prion disease presence, but also as a marker of disease progression. Moreover Snord3A levels were also elevated in scrapie infected mice, demonstrating Snord3A may be used as a diagnostic tool not only in genetic prion disease, but also in the transmissible prion etiologies. Future work will determine if this is also the case for sporadic CJD, for genetic forms associated with other PrP mutations. It will also be important to determine whether levels of Snord3A expression are also elevated in other neurodegenerative diseases such as Alzheimer’s or Parkinson’s disease. Determining whether elevation of Snord3A expression is specific for prion disease or otherwise also relates to other forms of neurodegeneration will help to decipher its role and mechanism of action in disease.

The fact that results in human blood for this marker were consistent with those obtained for the brains of transgenic mice carrying the same mutation are extremely important, and indicate that Snord3A can be used as a marker of disease progression in healthy genetic carriers of PrP mutations. If true for sporadic cases, Snord3A levels may be used to identify at risk individuals of all etiologies, and in the future, as a marker of treatment success. Snord3A, or in its other denomination U3 small nucleolar RNA, was shown to be part of the complex comprising fibrillarin and the survival motor neuron gene (SMN1) [41], [42], implicated in ALS pathogenesis via oxidative pathways [36]. On another note, other SnoRnas were recently recognized as mediators of oxidative and ER stress [25].

In addition to Snord3A, reduced expression of ALdh1, an enzyme related directly to the response to oxidative stress, was also found to be associated with humans suffering from E200K CJD. ALDH1 is the enzyme that degrades HNE, one of the most toxic byproducts of lipid peroxidation [26]. HNE was already implicated in oxidative stress pathological damage in other neurodegenerative diseases such as Alzheimer’s disease (AD), Parkinson’s disease (PD), and amyotrophic lateral sclerosis (ALS) [26], [43]. Reduced expression of ALdh1 in blood or even its mostly unchanged expression levels in brains (as found in our mice) may aggravate the damage of oxidative insults and thereby accelerate the disease process. While this transcript was not indicative in the TgMHu2ME199K brains, and thereby we could not study its relation to the kinetics of disease advance, it may still be tested further as a prion marker in patients and carriers.

As depicted in Figure 6, elevation of Snord3A may be associated with several steps of the UPR system, a pathway shown to play a role in several neurodegenerative conditions [44]. One possibility [Fig. 6 (1)] is that Snord3A expression is elevated following its interaction with the misfolded PrP aggregates, and that such elevation may result in the subsequent activation of ER stress and designated UPR arms. Otherwise [Fig. 6 (2)], Snord3A expression may be secondarily activated as a result of the ER stress induced by the accumulated proteins. A third possibility, not necessarily independent from the first ones, is that Snord3A interferes with the UPR pathway after the activation of ATF6 [Fig. 6(3)] and thereby inhibits BIP activation and its function as a protective chaperon. The fact that Snord3A was not activated in PrP ablated mice and cells [45], which were sensitive to oxidative insults in the form of copper ions, further implies its role is not a simple response to reduced oxidative protection, but rather a reaction to misfolded protein aggregation and its consequences vis a vis ER stress. In addition, our results show that if there is a role for the loss of PrP^C^ function in prion disease pathogenesis, it is not mediated by Snord3A.

In summary, our results indicate that Snord3A may constitute a disease dependent marker for several forms of prion disease. Further investigation in the direct function of Snord3A by ablation or overexpression of these transcripts in prion cells systems may generate important mechanistic data.

## Materials and Methods

### Ethical Statement

Animal studies were carried out in strict accordance with the recommendations in the Guide for the Care and Use of Laboratory Animals of the National Institutes of Health. The protocol was approved by the Committee on the Ethics of Animal Experiments of the Hebrew University Medical school). All surgery was performed under sodium pentobarbital anesthesia, and all efforts were made to minimize suffering.

Blood samples from patients, carriers and controls used in this study were obtained under the ethical permit granted to Prof Chapman from the Sheba Medical Center by the Israeli Ministry of health. Each person participating in this study signed an informed consent document.

### RNA Isolation

Human blood samples were collected into Tempus Blood RNA Tubes and stored at −20°C. RNA extractions were performed using the Tempus Spin RNA Isolation Kit (Life Technologies, CA USA) following manufacturer’s instructions. Mice brain samples were collected into tube containing RNA Save solution (Biological Industries, Israel) and stored at −80°C, Total RNA was isolated using TRI reagent (Sigma, Israel). Purified RNA samples were subjected to DNA digestion carried out with DNase I (Invitrogen). cDNA was prepared from 1 µg of total RNA using using High Capacity cDNA Reverse Kit (**Life Technologies**, CA USA), according to the manufacturer’s instructions.

Samples included in these studies were E200K patients at middle clinical stages (12), mean age 58, both female and male, as well as carriers and non-carriers of the mutation from the same families, both male and female, mean age of 51 (24). Youngest carrier included in the study as 38 and the older 67. Storage of blood samples for 6 months or 1 week before RNA extraction gave similar results.

### Microarray

Approximately 300 ng of RNA isolated from Blood samples from E200KCJD patients, healthy carriers and non-carrier family controls (5 individuals from each group) was hybridized to Human Gene 1.0 ST Arrays (Affymetrix, Santa Clara, CA.) according to the manufacturer’s protocol. The hybridized arrays were washed and stained on a GeneChip® Fluidics Station 450 and scanned with a GeneChip® Scanner 3000 7G. The CEL files were normalized using Robust Multichip Analysis (RMA) in the Partek Genomic Suite (Partek Inc., Saint Louis, MO.) ANOVA with nominal alpha value set to 0.05 was then used to determine the probe sets significantly different between the 3 groups of samples (patients; carriers and controls), Genes whose expression was up- or down-regulated in patients and carriers when compared to controls were identified.

#### Animals studies

Mice of different ages (TgMHu2ME199K mice [19]; PrP^0/0^ [45] mice crossed into C57b for 10 generations; and wt mice C57B from Harlan, Hebrew University, Jerusalem, Israel) were sacrificed and their brains separated for protein and RNA studies as described above. In some cases Cupric sulfate was administered to three months old mice for 75 days at a final concentration of 2 mM (300 ppm. All animal experiments were done according to institutional guidelines and National Institute of Health regulations. Groups in each experiment comprised 4–7 mice.

#### Prion infection

1- and 16-month-old C57BL/6 female mice (Harlan, Israel) were inoculated intracerebral (IC) injection with 50 µL or intraperitoneally (IP) with 85–100 µl of 1% Rocky Mountain Laboratory (RML) strain of prion-infected brain homogenate. Age-matched control mice (naïve) were inoculated with sterile PBS not containing infectious material. Mice were followed closely for disease signs until disease manifestation. At the terminal stage mice were sacrificed and their brains were collected for further analysis.

#### Real-Time PCR

RNA samples (10–15 subjects of each group) as described above were validated for the expression of Snord3A or ALDH1 by Quantitative RT-PCR. The reaction was carried out in 15 µl reactions containing 1 µl of cDNA, 0.3 µM of the appropriate primers (Sigma and Hylabs–UBC), and 7.5 µl of the Power SYBR Green master mix (**Life Technologies**, CA USA) ). Gene amplification was carried out using **Life Technologies**, CA USA Step One Plus Real-Time PCR System or the GeneAmp 7000 Sequence Detection. Cycling parameters were 95°C for 10 min and then 40 cycles of 95°C for 15 s, annealed/extended at 60°C for 1 min. Control reactions were run without reverse transcriptase and used as negative control. Measurements were performed in triplicates and Housekeeping gene transcript levels were used to normalize between samples. Data Assist v3.0 software (**Life Technologies**, CA USA) was used to determine the cycle crossover points (cycle threshold, *C*_t_) for each specimen.mRNA expression values were calculated using the comparative Ct (ΔΔCt) method.

The primers used were:

#### Human

*HPRT* , *5′-*AGA TGG TCA AGG TCG CAA GC*-3′* (forward); *5′-* CAT ATC CTA CAA CAA ACT TGT CTG GAA*-3′* (reverse); *SNORD3A* , *5′-* TCTGAACGTGTAGAGCACCGAA*-3′* (forward);*5′-*GACGGCAGTTGCAGCCAA *-3′* (reverse);*ALDH1A1*, *5′-* GTGAAGGCCGCAAGACAGG*-3′* (forward);*5′-*CCTGCACAGTAGCGCAATGTT*-3′* (reverse).

#### Mouse

*UBC*, *5′-CAG CCG TAT ATC TTC CCA GAC T-3′* (forward); *5′-CTC AGA GGG ATG CCA GTA ATC TA-3′* (reverse);*Actin beta*,*5′-*AAT CGT GCG TGA CAT TAA GG*-3′* (forward); *5′-*ACG CAA CTA AGT CAT AGT CC*-3′* (reverse); *RNU3 (snord3A)*,*5′-*CCGAAACCACGAGGACGAG*-3′* (forward); *5′-* CCTCTCACCCTCCCAAAGGA*-3′* (reverse).

#### Immunoblotting of mice brain homogenates

Brains from wt, TgMHu2ME199K, PrP ablated and scrapie RML infected mice were homogenate at 10% (W/V) in 10 mMTris-HCl, pH 7.4 and 0.3 M sucrose. Brain homogenate were assayed for total protein using a BCA assay (Pierce, Rockford, IL) to ensure that all samples loaded on the gel correspond to equal amounts of original homogenate. When designated samples were digested before immunoblotting with 40 ug/ml Proteinase K. P Samples were separated on SDS-PAGE. and immunoblotted with the designated antibodies, against ATF6 (sc-22799, Santa Cruz Biotechnology), BiP(sc-13968), Santa Cruz Biotechnology), or PrP (α PrP RTC [30]).

#### Statistical analysis

Statistical evaluation of significance differences was performed by using Student’s *t*-test and two Tails. *P*<0.05 was considered significant for our analysis.
