# Expression of Concern: Snord 3A: A Molecular Marker and Modulator of Prion Disease Progression

**DOI:** 10.1371/journal.pone.0316234

**Published:** 2024-12-18

**Authors:** The PLOS ONE Editors

Following the publication of this article [1], concerns were raised regarding the results presented in Figs 5 and 6. Specifically,

- Vertical discontinuities were detected in the following panels:
  ○ Fig 5, between lanes 2 and 3.
  ○ Fig 6A, between lanes 2 and 3.
  ○ Fig 6B, between lanes 2 and 3.
- In Fig 6B, there appear to be irregularities in the background surrounding the band in lane 5.

The corresponding author disagreed with PLOS’ findings of vertical discontinuities in Figs 5 and 6A. The underlying data provided for Fig 6A (S1 File) do appear to support the published results, but the resolution is too low to fully resolve the concerns. The corresponding author’s response did not resolve the issues raised with Fig 6B. The original data underlying the Fig 5 and 6B panels were not provided for editorial review. In the absence of these data, the concerns with Figs 5 and 6 cannot be fully resolved.

In light of the concerns raised the *PLOS ONE* Editors issue this Expression of Concern to notify readers of the above concerns and to relay the available data provided by the corresponding author.

## Supporting information

S1 File Original data underlying Fig 6A. (TIF)
