# Supplementary figures and images for: Expression of Concern: Snord 3A: A Molecular Marker and Modulator of Prion Disease Progression
Source: PLoS One. 2024 Dec 18;19(12):e0316234. doi: 10.1371/journal.pone.0316234 (PMC11654917; doi:10.1371/journal.pone.0316234)

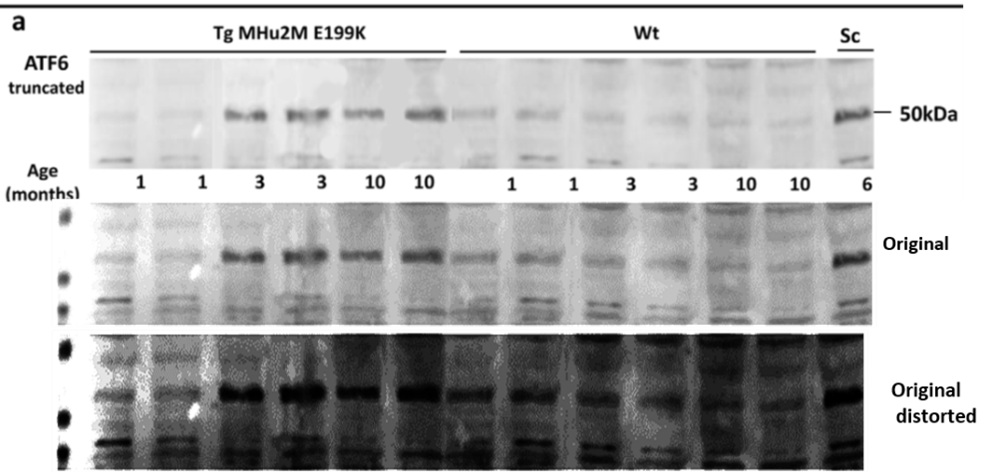

Supplement: S1 File — (TIF) [file pone.0316234.s001.tif]
